# Salivary Dysfunctions and Consequences After Radioiodine Treatment for Thyroid Cancer: Protocol for a Self-Controlled Study (START Study)

**DOI:** 10.2196/35565

**Published:** 2022-07-22

**Authors:** Clémence Baudin, Charlotte Lussey-Lepoutre, Alice Bressand, Camille Buffet, Fabrice Menegaux, Marine Soret, David Broggio, Céline Bassinet, Christelle Huet, Gemma Armengol, Laurence Leenhardt, Marie-Odile Bernier

**Affiliations:** 1 Ionizing Radiation Epidemiology Laboratory Institute for Radiological Protection and Nuclear Safety Fontenay-aux-Roses France; 2 Department of Nuclear Medicine Sorbonne University Pitié-Salpêtrière Hospital APHP Paris France; 3 Equipe Labellisée par la Ligue Contre le Cancer Paris Research Center Cardiovascular Institut National de la Santé et de la Recherche Médicale, University Paris City Paris France; 4 Amarexia France Paris France; 5 Thyroid and Endocrine Tumors Unit Groupe de Recherche Clinique Tumeurs Thyroïdiennes no. 16 Pitié-Salpêtrière Hospital APHP, Sorbonne University Paris France; 6 Department of General and Endocrine Surgery Pitié-Salpêtrière Hospital APHP Sorbonne University Paris France; 7 Biomedical Imaging Laboratory French National Centre for Scientific Research, Institut National de la Santé et de la Recherche Médicale Sorbonne University Paris France; 8 Internal Dose Assessment Laboratory Institut de Radioprotection et de Sûreté Nucléaire Fontenay-aux-Roses France; 9 Ionizing Radiation Dosimetry Laboratory Institut de Radioprotection et de Sûreté Nucléaire Fontenay-aux-Roses France; 10 Department of Animal Biology, Plant Biology and Ecology Faculty of Biosciences Universitat Autònoma de Barcelona Bellaterra, Catalonia Spain

**Keywords:** radioiodine, thyroid cancer, epidemiology, self-controlled study, salivary gland, lacrimal gland, dysfunction, protocol, epidemiology

## Abstract

**Background:**

Following radioiodine (^131^I) therapy of differentiated thyroid cancer, the salivary glands may become inflamed, leading to dysfunctions and decreases in patients’ nutritional status and quality of life. The incidence of these dysfunctions after ^131^I-therapy is poorly known, and no clinical or genetic factors have been identified to date to define at-risk patients, which would allow the delivered activity to be adapted to the expected risk of salivary dysfunctions.

**Objective:**

The aims of this study are to estimate the incidence of salivary dysfunctions, and consequences on the quality of life and nutritional status for patients after ^131^I-therapy; to characterize at-risk patients of developing posttreatment dysfunctions using clinical, biomolecular, and biochemical factors; and to validate a dosimetric method to calculate the dose received at the salivary gland level for analyzing the dose-response relationship between absorbed doses to salivary glands and salivary dysfunctions.

**Methods:**

This prospective study aims to include patients for whom ^131^I-therapy is indicated as part of the treatment for differentiated thyroid cancer in a Paris hospital (40 and 80 patients in the 1.1 GBq and 3.7 GBq groups, respectively). The follow-up is based on three scheduled visits: at inclusion (T0, immediately before ^131^I-therapy), and at 6 months (T6) and 18 months (T18) posttreatment. For each visit, questionnaires on salivary dysfunctions (validated French tool), quality of life (Hospital Anxiety and Depression scale, Medical Outcomes Study 36-Item Short Form Survey), and nutritional status (visual analog scale) are administered by a trained clinical research associate. At T0 and T6, saliva samples and individual measurements of the salivary flow, without and with salivary glands stimulation, are performed. External thermoluminescent dosimeters are positioned on the skin opposite the salivary glands and at the sternal fork immediately before ^131^I administration and removed after 5 days. From the doses recorded by the dosimeters, an estimation of the dose received at the salivary glands will be carried out using physical and computational phantoms. Genetic and epigenetic analyses will be performed to search for potential biomarkers of the predisposition to develop salivary dysfunctions after ^131^I-therapy.

**Results:**

A total of 139 patients (99 women, 71.2%; mean age 47.4, SD 14.3 years) were enrolled in the study between September 2020 and April 2021 (45 and 94 patients in the 1.1 GBq and 3.7G Bq groups, respectively). T6 follow-up is complete and T18 follow-up is currently underway. Statistical analyses will assess the links between salivary dysfunctions and absorbed doses to the salivary glands, accounting for associated factors. Moreover, impacts on the patients’ quality of life will be analyzed.

**Conclusions:**

To our knowledge, this study is the first to investigate the risk of salivary dysfunctions (using both objective and subjective indicators) in relation to organ (salivary glands) doses, based on individual dosimeter records and dose reconstructions. The results will allow the identification of patients at risk of salivary dysfunctions and will permit clinicians to propose a more adapted follow-up and/or countermeasures to adverse effects.

**Trial Registration:**

ClinicalTrials.gov NCT04876287; https://clinicaltrials.gov/ct2/show/NCT04876287

**International Registered Report Identifier (IRRID):**

DERR1-10.2196/35565

## Introduction

Approximately 10,600 new cases of thyroid cancer were diagnosed in France in 2018, and this incidence has increased by an average of 4.4% per year between 1990 and 2018 [1]. Although incidence rates are steadily increasing, mortality rates have declined slightly in recent decades, and thyroid cancer has an excellent prognosis with a 10-year survival rate of over 90% [1].

Standard treatment for differentiated thyroid cancer is thyroidectomy followed by radioiodine (^131^I) ablation [2]. This ^131^I complementary therapy aims to destroy thyroid remnant tissues, thus facilitating biological surveillance via the thyroglobulin dosage and reducing the risk of cancer relapse. This therapy also treats possible persistent or metastatic diseases, and enables assessment of disease extension through posttherapy scintigraphy. However, the usefulness of ^131^I-therapy is debated, especially for cancers with a low risk of recurrence because of their excellent survival rate, and the possibility of adverse effects of this therapy in the short, medium, and long term [3]. The salivary gland might be a site of inflammation after ^131^I-therapy owing to its ability to capture and concentrate iodine [4], and in particular radioactive iodine, which may be symptomatic in the acute phase, and can be followed by chronic salivary dysfunctions [5]. Such dysfunctions are defined as any quantitative or qualitative change in saliva production, such as a decrease in salivary secretion ranging from minor to severe hypofunction [6]. Nevertheless, saliva is crucial for the maintenance of buccal health. Notably, the incidence of salivary dysfunctions is still unclear, ranging from 2% to 67%, due to major methodological differences between studies, including the method and timing of identification of salivary dysfunctions [4].

Salivary dysfunctions may lead to an increased risk of inflammation and/or oral infection, a change in the taste of food, and difficulties in swallowing and digestion. Lacrimal gland dysfunctions (one of the diagnostic criteria for Sjögren syndrome) have also been reported after ^131^I-therapy, suggesting that some patients may develop simultaneous lacrimal and salivary gland dysfunctions in the years following ^131^I-therapy [7]. Quality of life after ^131^I-therapy requires special attention because of the good prognosis of differentiated thyroid cancer. In the context of ^131^I-therapy for thyroid cancer, few studies have assessed the long-term quality of life at 6 months or later after ^131^I-therapy [8-12], whereas it has been shown that salivary dysfunction assessed by a specific questionnaire may occur more than 6 months after therapy for 25% of patients [13]. Moreover, there are inconsistencies in the reported relationships between objective measures of salivary flow (sialometry) and subjective measures of salivary dysfunction (self-questionnaire) [6,11], reinforcing the need to assess salivary dysfunction by creating a composite criterion combining objective and subjective measures.

Additionally, the dose-response relationships between ^131^I-therapy and the incidence of salivary and lachrymal dysfunctions have been rarely studied, and the administered activity is typically used as a proxy for the dose absorbed by the salivary glands. However, the administered activity does not accurately reflect the dose received by the salivary glands due to the variation in iodine uptake ability of the salivary gland, which could be modified by the size of the thyroid remnant tissues or a potential iodine deficiency of the patient before treatment, along with the inherent interpatient variability affecting the entire biokinetics of iodine. The use of an adapted dosimetric method using thermoluminescent dosimeters and anthropomorphic phantoms would make it possible to better estimate the dose-response relationship by estimating the absorbed dose to the salivary gland.

Finally, while a strong interrelationship between epigenetic processes and genetic factors associated with dry mouth syndrome has been shown [14], the potential existence of individual genetic sensitivity to ^131^I in mediating the relationship between ^131^I exposure and salivary dysfunctions is important to study, especially as specific epigenetic changes could lead to increased sensitivity to ionizing radiation [15]. Other risk factors of salivary dysfunctions in the context of thyroid cancer treated by ^131^I-therapy are poorly understood. Only one study investigated the relationship between salivary flow and age, gender, or pathological tumor-node-metastasis (TNM) staging, but did not show any significant relationship, potentially owing to a lack of statistical power due to the small number of patients included (N=67) [11], emphasizing the importance of setting up a study with a large number of patients. Increased knowledge of these factors would help to identify the patients at risk of developing salivary dysfunctions, which could possibly help to adapt the treatment and follow-up accordingly.

In this context, the Salivary dysfuncTions After Radioiodine Treatment (START) study was launched in September 2020, with the following objectives: (1) to estimate the mid- and long-term incidence of salivary dysfunctions in patients with thyroid cancer treated with ^131^I, using objective, subjective, and mixed criteria; (2) to highlight risk factors of posttreatment salivary dysfunctions using clinical, pathological, biomolecular, and biochemical factors; (3) to validate a dosimetric method to calculate the dose absorbed by the salivary gland; (4) to estimate the dose-response relationship between exposure of the salivary glands to ^131^I and salivary dysfunctions; and (5) to investigate the consequences of salivary dysfunctions on the quality of life and nutritional status of patients.

## Methods

### Study Design

START is a prospective self-controlled study that includes patients with thyroid cancer who underwent thyroidectomy and are candidates for complementary ^131^I-therapy. This research program aims to enroll 120 patients from the nuclear medicine department of Pitié-Salpêtrière Hospital (Paris, France), divided into two groups of 40 and 80 patients treated with ^131^I activity of 1.1 GBq and 3.7 GBq, respectively, thus expecting a high number of patients with salivary dysfunctions to provide good statistical power for the analyses.

**Figure 1:**
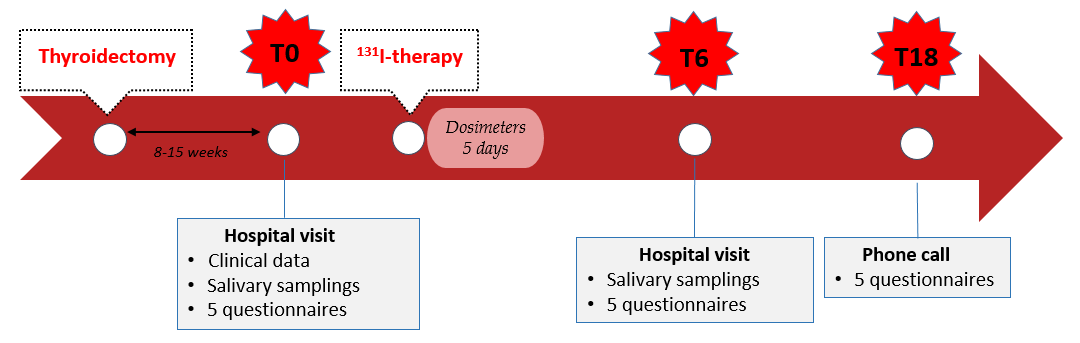
Timeline summarizing the study's outline. T0: enrollment; T6: 6 months after radioiodine (^131^I) therapy; T18: 18 months after radioiodine therapy.

All consecutive patients awaiting ^131^I-therapy are systematically invited to participate in the study. They are enrolled just prior to the ^131^I capsule administration (ie, 1 to 3 months after thyroidectomy), and have three follow-up points: at enrollment (T0), and at 6 months (T6) and 18 months (T18) after ^131^I-therapy (Figure 1).

### Inclusion and Exclusion Criteria

To be included, patients must have undergone thyroidectomy for the treatment of a differentiated thyroid cancer, be a candidate for complementary ^131^I-therapy, be over 18 years of age, be willing to participate in the study, and have signed a consent form. Patients who have previously been treated with ^131^I or who are likely to be treated with multiple ^131^I therapies within 18 months of inclusion are excluded.

### Ethical Considerations

This study will be conducted in accordance with the Declaration of Helsinki (amended at the 64th World Medical Association General Assembly, Fortaleza, Brazil, October 2013) and in accordance with the principles of “Good Clinical Practice” and the Medical Research Involving Human Subjects Act (WMO) [16].

Approvals from the local ethical committee have been received (Comité de Protection de Personnes Sud Mediterranée III, ID 20.01.24.56149; and Agence Nationale de Sécurité des Médicaments, ID 2020-A00208-31). The protocol is registered and will be posted on the ClinicalTrials.gov public website under the number NCT04876287.

Patients included in the study must sign a consent form in which they certify that they have understood the objectives and procedures of the research in which they will participate. They certify that they have had time to think and ask questions to come to an informed decision to participate, and they are aware that this study is not mandatory in the context of their therapy and that they can withdraw at any time.

### Conduct of the Study

#### Schedule

The START study is expected to be carried out from September 2020 until October 2022, including three measuring points for each patient: at enrollment, and at 6 and 18 months later.

#### Before Enrollment

The investigating physician in the nuclear medicine department enables first contact with patients during the postsurgery consultation. During this first visit, the physician introduces the START study and its objectives to the patients for whom ^131^I-therapy is planned, informs them about the nature of the constraints and the expected benefits of the research, and answers all of the patient’s questions. The physician ensures that patients meet the inclusion criteria. If the inclusion criteria are fulfilled, a study information leaflet detailing the protocol is given to the patient with the consent form.

#### Enrollment Visit (T0)

The enrollment visit occurs when an eligible patient has given consent to participate in the study. This visit takes place at the hospital during the consultation prior to the complementary ^131^I-therapy (on the day of treatment, approximately 2 months after surgery) in a face-to-face manner with a clinical research associate. Clinical and personal data and saliva samples are collected, self-administered questionnaires are provided to patients (see Data Collection section below and Multimedia Appendix 1), and the patients are equipped with thermoluminescent dosimeters (see Dosimetry section below) just before administration of the ^131^I-therapy. The dosimeters will be removed by the physician 5 days later, immediately before the posttherapy scan.

#### Six-Month Follow-up (T6)

The 6-month follow-up takes place during the posttherapy consultation at the Thyroid and Endocrine Tumors Unit, Institute of Endocrinology (E3M), Pitié-Salpêtrière Hospital (Paris, France), in a face-to-face manner with a clinical research associate. During this visit, clinical and personal data collection, saliva samples, and self-questionnaires are again carried out

#### Eighteen-Month Follow-up (T18)

Patients are called by phone to answer the self-questionnaires with a clinical research associate.

### Data Collection

All data are collected using paper versions, and are then entered and recorded on a secure server (Table 1).

All questionnaires are completed at each measuring point of the study for each patient. Questionnaires have been selected on the basis of ease and speed of administration by an interviewer, peer-validated, and on the fact that they are the most widely used questionnaires in this type of research (see Multimedia Appendix 1).

Saliva sampling has been performed according to a standardized methodology (see Multimedia Appendix 2).

**Table 1:** Summary of data collection.

Data type	Qualitative data	Quantitative data
Thyroid cancer information (collected only at the enrollment visit)	Size of the postsurgery remnant, tumor histology, pTNM^a^ staging, Tg^b^ stimulation protocol, family history of thyroid cancer, patient history of cancer or comorbidities, prescribed activity of ^131^I (1.1 or 3.7 GBq)	Surgery to therapy duration (months)
Clinical data	Self-rated menopausal status, all medications used during the last 3 months, tobacco and alcohol consumption, self-palpation of the salivary glands (normal, painful, or swollen), self-reported oral carries or infections, observed cracked lips, self-reported use of a saliva substitute, sialagogue	Age, height, weight
Questionnaires	Salivary complaints questionnaire; eye dryness, OSDI^c^ questionnaire; questions about nutrition; anxiety and depressive symptoms (HAD^d^ scale)	Physical and mental composite scores about quality of life (MOS SF-36^e^)
Saliva samples	Not applicable	Weight (precision 0.01 mg), volume (precision 0.01 mL), electrolyte concentrations (mmol/L: sodium, potassium, chloride, amylase, and total protein composition), genetic and epigenetic variant proportions

^a^pTNM: pathological tumor-node-metastasis.

^b^Tg: thyroglobulin.

^c^OSDI: Ocular Surface Disease Index.

^d^HAD: Hospital Anxiety and Depression.

^e^MOS SF-36: Medical Outcome Study Short Form 36 items.

### Dosimetry

**Figure 2:**
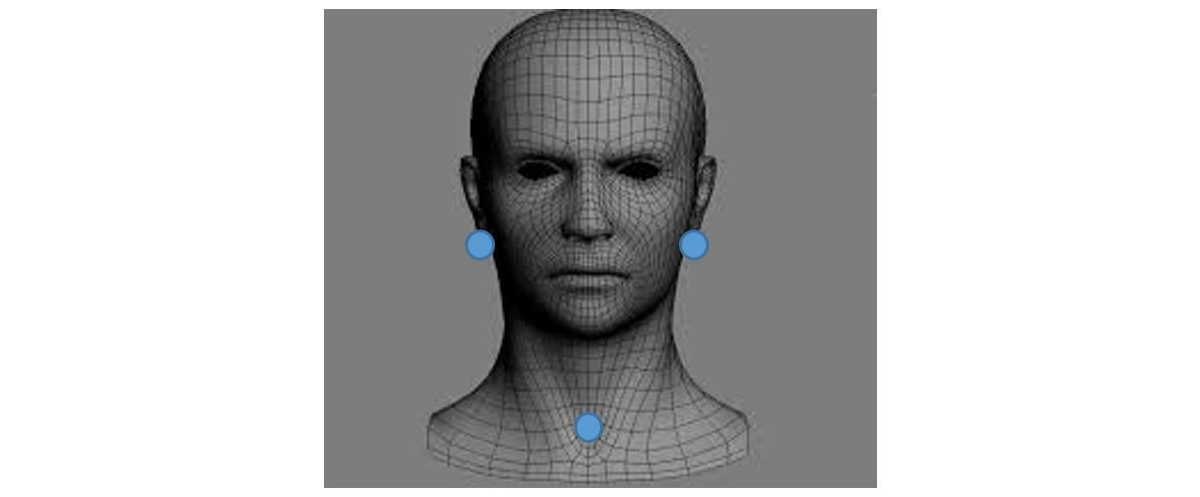
Thermoluminescent dosimeter position.

Immediately before therapy, three thermoluminescent dosimeters (^7^LiF: Mg,Ti, Thermo Scientific DXT-RAD dosimeter model: DXT-700, provider: APVL) are placed under each earlobe (at the salivary glands level) and at the sternal fork level, protected by an adhesive plastic film (Figure 2). They are removed 5 days later, just before the posttherapy scan.

Subsequently, the dose received specifically by the salivary glands will be estimated from the dosimeter readings. This requires the development of a specific calibration protocol that allows relating the dosimeter readings to the cumulative activity in the salivary glands and thyroid residues, using computational human models [17]. To validate this method, the response of thermoluminescent dosimeters to iodine does not have to deviate significantly from their response under standard calibration conditions.

### Study Endpoints

#### Primary Endpoint

The primary endpoint is a composite criterion combining objective (salivary flow dysfunctions with and/or without stimulation) and subjective (reporting discomfort, pain in the parotid area, difficulty swallowing, or eye dryness) criteria at T6. Salivary flow dysfunctions are defined as an unstimulated salivary flow <0.2 mL/min and/or a stimulated salivary flow <0.7 mL/min [18,19].

#### Secondary Endpoints

Secondary endpoints are defined as follows: change in quality-of-life score between T0, T6, and T18; change in nutrition between T0, T6, and T18; change in chemical saliva composition between T0 and T6; change in anxiety and depression symptoms between T0, T6, and T18; genetic and/or epigenetic variant identification that may modify the risk of salivary dysfunctions after ^131^I-therapy; identification of individual factors associated with salivary dysfunctions at T6 and T18; and dose-response analysis between the ^131^I-dose received precisely at the salivary glands and salivary dysfunctions at T6 and T18.

### Statistical Analyses

#### Sample Size Calculation

A minimum of 120 patients is required to complete the START study. This number is based on a statistical power of 80% with an α risk of 5%, and with theoretical percentages from the literature of salivary dysfunctions of 30% and 60% for the 1.1 GBq and 3.7 GBq therapy groups, respectively [4,20,21]. Thus, the number of patients required is distributed as follows: 40 and 80 patients in the groups treated with delivered activities of 1.1 GBq and 3.7 GBq, respectively. Furthermore, to consider potential losses during follow-up, a final number of 130 patients is required.

#### Descriptive Analyses

The description of the population (means, SDs, and percentages) at baseline (before ^131^I-therapy) is presented in this paper. Comparison tests have been performed (*t* test and *χ*^2^ tests) between the two groups of patients (1.1 GBq and 3.7 GBq groups) at T0. This is based on medical and individual data collected in the questionnaire, the ^131^I dose received precisely at the salivary glands in comparison with the delivered activity, saliva flow descriptions, and responses to the questionnaires.

#### Planned Analyses

Paired comparison tests will be used to assess differences in data between T0 and T6. Correlations, principal component analyses, and multiple correspondence analyses will be used to study the factors (objective and subjective) common to patients with salivary dysfunctions after ^131^I-therapy.

All factors significant at a 10% threshold in univariate analyses using different regression models (Textbox 1) will then be entered into multivariate models to measure the risk of developing salivary dysfunctions or other outcomes, adjusted for risk factors previously identified.

The mixed regression models, adjusted for confounders, performed to investigate the temporal evolution for the different outcomes, will be set up using a random intercept for each patient with a first-order autoregressive covariance structure to account for repeated measurements.

The different scores to the questionnaires for quality of life, nutrition, and symptoms of depression and anxiety assessments are calculated and classes are created according to the recommendations for each questionnaire and for each visit (T0, T6, and T18).

**Synthesis of the planned analyses.**

**Outcomes**

- Categorical

Dry mouth sensation after treatment, dry eye (Ocular Surface Disease Index), Hospital Anxiety and Depression (HAD) anxiety scale, HAD depression scale, changes in nutrition, composite outcome (sum of dysfunctions)

- Continuous

Unstimulated saliva flow (mL/min), stimulated saliva flow (mL/min), saliva pH, electrolyte concentrations in saliva (mmol/L), physical composite score (Medical Outcome Study Short Form 36 items [MOS SF-36]), mental composite score (MOS SF-36), genetic and/or epigenetic variants

**Models and tests**

- Categorical

*χ*^2^, logistic regression, Poisson regression

- Continuous

Correlations, *t* test, linear regressions, mixed model

**Factor of interest**

Dose received by the salivary glands, administered dose

**Adjustment factors to be tested**

Gender, self-rated menopausal status, age, BMI, histology, thyroid remnant tissue, pathological tumor-node-metastasis staging, thyroglobulin stimulation protocol, salivary comorbidities, medication intake, tobacco and alcohol consumption, surgery to therapy duration (months), family history of thyroid cancer

All statistical analyses will be performed using SAS statistical software for Windows (SAS Institute, Cary, NC). An α level of .05 will be accepted as significant.

## Results

Patient enrollment took place from September 2020 to April 2021, with a total of 139 patients enrolled, including 45 and 94 patients treated with 1.1 and 3.7 GBq ^131^I, respectively.

Characteristics of the study participants are displayed in Table 2. The START sample is composed of a large majority of women, with an average age of 47 years (range 18.70-81.80 years). All patients underwent total thyroidectomy with a diagnosis of differentiated thyroid cancer confirmed by pathological examination; the highest proportion in the TNM classification was Tx-T2, followed by Nx-N0, N1, T3, and T4, including approximately 47% of patients with residual thyroid tissue. The ^131^I-therapy was performed an average 4.11 (SD 3.91) months after the surgery (range 1-35 months). The majority of the included patients were treated for a papillary thyroid cancer.

Concerning the saliva samples, the mean pH was 7.34 (SD 0.48; range 6.00-8.50), with flow rates of 0.76 (SD 0.46; range 0.04-3.00) mL/min and 2.13 (SD 0.88; range 0.40-4.82) mL/min for unstimulated and stimulated saliva, respectively.

Statistically significant differences between the 1.1 and 3.7 GBq treated groups were found in saliva pH and nonstimulated saliva volume.

At the enrollment visit, among all 139 patients, 19 (13.6%) patients had suspicious or obvious symptoms of depression, whereas 67 (48.2%) patients had suspicious or obvious symptoms of anxiety according to the Hospital Anxiety Depression scale (see Table S1 in Multimedia Appendix 3).

When asked if patients experienced changes after thyroid removal surgery, 25 (18.0%) had the sensation of a dry mouth, 48 (34.5%) said they drink more often, and 27 (19.4%) were eating less salty foods.

Regarding lifestyle habits, among the 139 patients, 95 (68.3%) had never smoked, compared to 28 (20.1%) exsmokers and 16 (11.5%) current smokers; 87 (62.6%) never drink alcohol, compared to 45 (32.4%) being occasional drinkers (1-7 drinks/week) and 7 (5.0%) being regular drinkers (7-14 drinks/week) (see Table S1 in Multimedia Appendix 3).

Regarding radioiodine activity, the average doses recorded by the thermoluminescent dosimeters are presented in Table 3. Preliminary results based on the numerical calibration of the dosimeters in terms of cumulated activity provide absorbed doses to the salivary glands after application of the S-factor (self-dose) for salivary glands. There were 43 and 127 patients for which dosimeters were readable in the 1.1 GBq group and 3.7 GBq group, respectively. The doses to the salivary glands were approximately 3-times higher in the 3.7 GBq group than in the 1.1 GBq group (Table 3). For the whole group, the dose to the salivary glands per unit administered activity was 0.63 (SD 0.24) Gy/MBq. The ratio of left/right salivary glands cumulated activity was 0.97 (SD 0.15).

**Table 2:** Characteristics of the study population.

Characteristics			1.1 GBq group (n=45)		3.7 GBq group (n=94)		Total (N=139)		*P* value^a^
**Gender, n (%)**									.24
	Women	35 (78)		64 (68)		99 (71.2)			
	Men	10 (22)		30 (32)		40 (28.8)			
Age (years), mean (SD)			47.16 (13.86)		47.02 (14.36)		47.07 (14.15)		.96
BMI, mean (SD)			26.88 (5.93)		27.14 (6.12)		27.06 (6.04)		.82
**Histology, n (%)**									.02
	Follicular	2 (4)		18 (19)		20 (14.4)			
	Papillary	43 (96)		76 (81)		119 (85.6)			
**pTNM^b^ staging, n (%)**									<.001
	Tx-T2	44 (100)		59 (63)		103 (74.6)			
	T3	0 (0)		33 (35)		33 (23.9)			
	T4	0 (0)		2 (2)		2 (1.5)			
	Nx-N0	38 (84)		40 (43)		78 (56.1)			
	N1	7 (16)		54 (57)		61 (43.9)			
**TSH^c^ elevation protocol, n (%)**									<.001
	L-thyroxin replacement stop	0 (0)		49 (52)		49 (35.2)			
	rTSH^d^	45 (100)		45 (48)		90 (64.8)			
**Thyroid remnant tissue, n (%)**									.07
	No	29 (64)		45 (48)		74 (53.2)			
	Yes	16 (36)		49 (52)		65 (46.8)			
**Family history of thyroid cancer, n (%)**									.66
	No	37 (82)		80 (85)		117 (84.2)			
	Yes	8 (18)		14 (15)		22 (15.8)			
**History of systemic disease, n (%)**									.19
	No	31 (69)		66 (70)		97 (69.8)			
	Type 2 diabetes	3 (7)		2 (2)		5 (3.6)			
	Dyslipidemia	3 (7)		3 (3)		6 (4.3)			
	Diagnosed hypertension	6 (13)		22 (23)		28 (20.1)			
	Sjögren syndrome	0 (0)		1 (1)		1 (0.7)			
	Brain tumors	1 (2)		0 (0)		1 (0.7)			
	Other	1 (2)		0 (0)		1 (0.7)			
**History of salivary dysfunctions, n (%)**									.15
	No	41 (91)		91 (97)		132 (95.0)			
	Yes	4 (9)		3 (3)		7 (5.0)			
Delay between surgery and RAI^e^ therapy (months), mean (SD)			4.98 (5.15)		3.69 (3.09)		4.11 (3.91)		.07
Unstimulated saliva flow (mL/min), mean (SD)			3.13 (1.82)		4.12 (2.47)		3.8 (2.32)		.02
Stimulated saliva flow (mL/min), mean (SD)			9.65 (3.18)		11.12 (4.83)		10.65 (4.41)		.07
**Dry mouth sensation after treatment, n (%)**									.67
	Yes	9 (20)		16 (17)		25 (18.0)			
	No	36 (80)		78 (83)		114 (82.0)			
**Saliva pH, mean (SD)**			7.2 (0.42)		7.41 (0.50)		7.34 (0.48)		.02
**Dry eye (OSDI^f^), n (%)**									.29
	Normal	34 (79)		78 (86)		112 (83.6)			
	Light	3 (7)		8 (9)		11 (8.2)			
	Mild	4 (9)		2 (2)		6 (4.5)			
	Severe	2 (5)		3 (3)		5 (3.7)			

^a^*P* values are based on the *t* test for continuous variables or the *χ*^2^ test for categorical variables.

^b^pTNM: pathological tumor-node-metastasis.

^c^TSH: thyroid-stimulating hormone.

^d^rTSH: recombinant thyroid-stimulating hormone.

^e^RAI: radioactive iodine.

^f^OSDI: Ocular Surface Disease Index.

**Table 3:** Dosimetry description.

Variables	1.1 GBq group (n=45)	3.7 GBq group (n=94)
Recorded doses at the left earlobe [Hp(0.07), mSv], mean (SD)	30.07 (9.81)	104.38 (35.43)
Recorded doses at the right earlobe [Hp(0.07), mSv], mean (SD)	30.74 (8.88)	107.72 (42.66)
Recorded doses at the sternal fork [Hp(0.07), mSv], mean (SD)	49.88 (31.47)	173.33 (94.2)
Absorbed doses to the salivary glands (mGy), mean (SD)^a^	702.00 (310.00)	2316.00 (784.00)

^a^N=127.

Further results should be published in 2022. These will be based on comparisons of data from questionnaires and saliva samples between T0 and T6. Individual factors associated with salivary dysfunctions will be presented, as well as the dose-response relationship between absorbed doses to the salivary glands and salivary dysfunctions at T6 and T18.

## Discussion

Since many patients treated with ^131^I for thyroid cancer report salivary disorders, interfering with their quality of life, the START study was launched to detect and evaluate early and mid-term radiation-induced toxicity after ^131^I-therapy based on a prospective self-controlled study of patients with thyroid cancer. As an original multidisciplinary approach, the START study was designed to combine both objective and subjective parameters of quality of life with clinical and genetic information based on precise dosimetry, which better reflects the heterogeneity of the dose absorbed by the salivary glands. The use of several dosimeters makes it possible to discriminate the contribution coming from the thyroid remnants, after calibration under representative geometrical conditions. This approach further avoids having to perform several imaging examinations for the patients, and to integrate, de facto, the temporal variations of the activity in the salivary glands.

Moreover, this study will be the first to evaluate the genetic and epigenetic variants involved in salivary dysfunctions in patients treated with ^131^I, which may help to understand some of the biological mechanisms involved in radiation-induced sensitivity. These results should help to better estimate the individualized risk of long-term salivary dysfunctions after ^131^I-therapy, and thus allow the consideration of potential adverse effects in the choice of treatment.

As the study is still ongoing, this paper presents the protocol and objectives of the START study, as well as descriptive analyses of the included population and the doses recorded by the dosimeters. It is noteworthy that the sex ratio in the study reflects that of the population treated in nuclear medicine departments, which allows for an accurate estimation of the incidence of salivary disorders [22]. Regarding dosimetry, it can be observed that the recorded doses are highly correlated to the administered activities, with a strong earlobes left/right symmetry.

The START study is carried out in collaboration with the Pitié-Salpêtrière Hospital Group, which is the largest European center for the treatment of thyroid cancer, with 10 to 12 patients per week treated with ^131^I-therapy in the nuclear medicine department. The main limitation of this study is the potentially small number of patients presenting posttherapy salivary dysfunctions. However, the number of subjects to be included was calculated on the basis of 80% statistical power with a first-order risk of 5% and percentages of salivary dysfunctions estimated from the literature [4,20,21]. Nevertheless, salivary complications following ^131^I-therapy have been poorly studied, and the incidence rate is not very well estimated.

This study will help to deepen knowledge on the risks of salivary dysfunctions after ^131^I-therapy, as well as provide a better understanding of involved genetic factors. The findings will help target patients at risk of developing salivary dysfunctions and possibly adapt the treatment, thus improving the quality of life and nutritional status of patients with thyroid cancer.

## Appendix

Multimedia Appendix 1 Questionnaires.

Multimedia Appendix 2 Saliva sampling.

Multimedia Appendix 3 Table S1.

## Abbreviations

^131^I: radioiodine
START: Salivary dysfuncTions After Radioiodine Treatment
T0: baseline enrollment
T6: 6-month follow-up
T18: 18-month follow-up
TNM: tumor-node-metastasis staging
