# Supplementary material for: Salivary Dysfunctions and Consequences After Radioiodine Treatment for Thyroid Cancer: Protocol for a Self-Controlled Study (START Study)
Source: JMIR Res Protoc. 2022 Jul 22;11(7):e35565. doi: 10.2196/35565 (PMC9356333; doi:10.2196/35565)
Supplement: Multimedia Appendix 1 [file resprot_v11i7e35565_app1.doc]

## Appendix 1: Questionnaires

##### Salivary complaints questionnaire

This questionnaire was developed and validated in French by E. Moreddu (2017) [1]. It asks about the feeling of tension, pain, or swelling (discomfort) in the parotid gland, dry mouth or xerostomia, and actions taken to relieve these pains, using verbal items.

###### Quality of life questionnaire – MOS SF-36

The Medical Outcome Study Short Form (MOS SF-36) was developed by Ware and Sherbourne (1992) from the 149-items MOS questionnaire [2]. It is a multidimensional and generic scale that assesses health status independently of causative disease, gender, age, and treatment. This scale can be administered as a self- or hetero-questionnaire, and requires only 5 to 10 minutes. Its 36 items assess 8 dimensions: physical activity, role physical; physical pain; general health, vitality, social function, emotional feelings, and mental health, as well as a special dimension, namely the evaluation of perceived health compared to one year earlier. Each item is translated into a scale from 0 to 100. The lower the score, the greater the disability.

##### Eye dryness – OSDI© questionnaire

The OSDI© questionnaire assesses dry eye symptoms and how they affect vision-related functioning during the last week of life using 12 questions [3]. The questionnaire is composed of 3 subscales: ocular symptoms, vision-related functioning and environmental triggers, with responses ranging from "never" to "all the time." A final score is calculated from 0 to 100, with scores of 0 to 12 representing normality, 13 to 22 representing mild dry eye, 23 to 32 representing moderate dry eye and over 33 representing severe dry eye [4].

##### Questions about nutrition

Self-reported body weight and height are collected during the questionnaire. The evaluation of malnutrition corresponds to a weight loss greater than 5% of the usual weight in less than one month, or greater than 10% of the usual weight in six months [5].

Additionally, a visual analog assessment (VAS) of food intake is administered [6]. The VAS is the standard reference tool for grading current versus usual food intake on a scale of 0 to 10, i.e. from "nothing at all" to "as usual". VAS are a useful and practical tool for recording and assessing symptom control and changes over time in cancer patients, by providing quantitative data. They can therefore be used by the patient or all members of the care team. VAS scores correlate well with objective measures such as weight. Therefore, they have been validated to screen for potential nutritional difficulties in patients treated for head and neck cancer [7]. Individual questions about meal frequency and type of food were also asked.

##### Anxiety and depressive symptoms questionnaire – HAD scale

The Hospital Anxiety and Depression (HAD) scale is an instrument screening for anxiety and depressive dysfunctions. It includes 14 items rated from 0 to 3. Seven questions relate to anxiety and seven others to depression, thus making it possible to obtain two scores (maximum score for each score = 21). To screen for anxiety and depressive symptomatology, the following interpretation can be proposed for each score: 7 or less = no symptomatology, 8 to 10 = suspected symptomatology, 11 and more: definite symptomatology [8].

1. Moreddu, E.; Baumstarck-Barrau, K.; Gabriel, S.; Fakhry, N.; Sebag, F.; Mundler, O.; Chossegros, C.; Taïeb, D. Incidence of Salivary Side Effects after Radioiodine Treatment Using a New Specifically-Designed Questionnaire. *Br. J. Oral Maxillofac. Surg.* **2017**, *55*, 609–612, doi:10.1016/j.bjoms.2017.03.019.

2. Ware, J.E.; Sherbourne, C.D. The MOS 36-Item Short-Form Health Survey (SF-36). I. Conceptual Framework and Item Selection. *Med. Care* **1992**, *30*, 473–483.

3. Schiffman, R.M.; Christianson, M.D.; Jacobsen, G.; Hirsch, J.D.; Reis, B.L. Reliability and Validity of the Ocular Surface Disease Index. *Arch. Ophthalmol. Chic. Ill 1960* **2000**, *118*, 615–621, doi:10.1001/archopht.118.5.615.

4. Grubbs, J.R.; Tolleson-Rinehart, S.; Huynh, K.; Davis, R.M. A Review of Quality of Life Measures in Dry Eye Questionnaires. *Cornea* **2014**, *33*, 215–218, doi:10.1097/ICO.0000000000000038.

5. Haute Autorité de Santé Diagnostic de la dénutrition de l’enfant et de l’adulte 2019.

6. Stubbs, R.J.; Hughes, D.A.; Johnstone, A.M.; Rowley, E.; Reid, C.; Elia, M.; Stratton, R.; Delargy, H.; King, N.; Blundell, J.E. The Use of Visual Analogue Scales to Assess Motivation to Eat in Human Subjects: A Review of Their Reliability and Validity with an Evaluation of New Hand-Held Computerized Systems for Temporal Tracking of Appetite Ratings. *Br. J. Nutr.* **2000**, *84*, 405–415, doi:10.1017/s0007114500001719.

7. Macqueen; Frost Visual Analogue Scales: A Screening Tool for Assessing Nutritional Need in Head and Neck Radiotherapy Patients. *J. Hum. Nutr. Diet.* **1998**, *11*, 115–124, doi:10.1046/j.1365-277X.1998.00088.x.

8. Snaith, R.P.; Zigmond, A.S. The Hospital Anxiety and Depression Scale. *Br. Med. J. Clin. Res. Ed* **1986**, *292*, 344.
