# Supplementary material for: Salivary Dysfunctions and Consequences After Radioiodine Treatment for Thyroid Cancer: Protocol for a Self-Controlled Study (START Study)
Source: JMIR Res Protoc. 2022 Jul 22;11(7):e35565. doi: 10.2196/35565 (PMC9356333; doi:10.2196/35565)
Supplement: Multimedia Appendix 2 [file resprot_v11i7e35565_app2.doc]

## Appendix 2: Saliva sampling

At both T0 and T6, total saliva before stimulation and after stimulation of the salivary glands is collected using standard methods [1].

Briefly, patients are asked not to eat, drink, chew gum, brush their teeth, or smoke for at least 60 minutes prior to saliva collection. Saliva is sampled under quiet and restful conditions, in an upright position, following these steps:

1. Unstimulated whole saliva is collected for five minutes by spitting out into a sterile graduated container (test tube);
2. Patients are asked to chew a gum until it softens (30 s to 1 min) to stimulate salivary glands (saliva remaining in the mouth at this time is evacuated before starting the new following saliva collection);
3. Stimulated whole saliva is collected in a similar manner as the unstimulated collection, for five minutes, into a new test tube. During this collection, the patient should continue chewing the gum for 5 minutes, alternating chews on each side and spitting out saliva.

Saliva samples have to be taken at the same time of the day between 9am and 1pm for both T0 and T6. After collections, within a maximum of 12 hours, saliva samples are stored at -80°C.

The chemical composition (sodium, potassium, chloride, amylase, and total protein composition) of saliva will be analyzed by the Biology Laboratory of the Pitié-Salpêtrière Hospital.

The expression of exosomal miRNA/SnoRNA in the saliva of patients having developed or not salivary dysfunctions will be compared between before and after ^131^I-therapy in the Faculty of Biosciences, Universitat Autònoma de Barcelona. The isolation of salivary exosomes will be performed using the Total Exosome Isolation kit (Invitrogen). Exosomal RNAs will be extracted (Total Exosome RNA and Protein Isolation Kit (Invitrogen)), and hybridized on Affimetrix chips (Affimetrix GeneChip™ miRNA 4.0 Array). A search for miRNA/SnoRNA signatures predictive of the development of salivary dysfunctions will be conducted by comparing salivary miRNome/SnoRNAs before and/or after ^131^I-therapy of patients with and without these dysfunctions using the EMts_2PCA method [2].

1. Navazesh, M. Methods for Collecting Saliva. *Ann. N. Y. Acad. Sci.* **1993**, *694*, 72–77, doi:10.1111/j.1749-6632.1993.tb18343.x.

2. Ugolin, N.; C, O.; E, L.; N, B.; P, H.; M, S.; S, C. Strategy to Find Molecular Signatures in a Small Series of Rare Cancers: Validation for Radiation-Induced Breast and Thyroid Tumors. *PloS One* **2011**, *6*, doi:10.1371/journal.pone.0023581.
