# Supplementary material for: Salivary Dysfunctions and Consequences After Radioiodine Treatment for Thyroid Cancer: Protocol for a Self-Controlled Study (START Study)
Source: JMIR Res Protoc. 2022 Jul 22;11(7):e35565. doi: 10.2196/35565 (PMC9356333; doi:10.2196/35565)
Supplement: Multimedia Appendix 3 [file resprot_v11i7e35565_app3.doc]

## Appendix 3: Table S1

| **Variables** | **Categories** | **1.1 GBq group** | **3.7 GBq group** | **Total** | ***P^c^*** |
| --- | --- | --- | --- | --- | --- |
| **Physical Composite score** (MOS SF-36)*^b^* | | 28.72 (1.28) | 28.64 (1.19) | 28.67 (1.21) | .71 |
| **Mental Composite score** (MOS SF-36)*^b^* | | 22.78 (1.32) | 22.65 (1.88) | 22.69 (1.71) | .67 |
| **HAD anxiety scale***^a^* | |  |  |  | .40 |
|  | Definite case | 8 (17.78) | 23 (24.47) | 31 (22.30) |  |
|  | Doubtful case | 10 (22.22) | 26 (27.66) | 36 (25.90) |  |
|  | No symptom | 27 (60.00) | 45 (47.87) | 72 (51.80) |  |
| **HAD depression scale***^a^* | |  |  |  | .56 |
|  | Definite case | 2 (4.44) | 9 (9.57) | 11 (7.91) |  |
|  | Doubtful case | 3 (6.67) | 5 (5.32) | 8 (5.76) |  |
|  | No symptom | 40 (88.89) | 80 (85.11) | 120 (86.33) |  |
| **Menopausal women***^a^* | |  |  |  |  |
|  | No | 24 (68.57) | 40 (62.50) | 64 (64.65) | .55 |
|  | Yes | 11 (31.43) | 24 (37.50) | 35 (35.35) |  |
| **Alcohol consumption***^a^* | |  |  |  | .55 |
|  | No | 30 (66.67) | 57 (60.64) | 87 (62.59) |  |
|  | Occasional | 12 (26.67) | 33 (35.11) | 45 (32.37) |  |
|  | Regular | 3 (6.67) | 4 (4.26) | 7 (5.04) |  |
| **Smoking habits***^a^* | |  |  |  | .62 |
|  | Non smoker | 33 (73.33) | 62 (65.96) | 95 (68.35) |  |
|  | Current smoker | 5 (11.11) | 11 (11.70) | 16 (11.51) |  |
|  | Ex-smoker | 7 (15.56) | 21 (22.34) | 28 (20.14) |  |
| **Changes in drinking since thyroid removal surgery***^a^* | |  |  |  | .34 |
|  | No change | 31 (68.89) | 56 (59.57) | 87 (62.59) |  |
|  | More rarely | 2 (4.44) | 2 (2.13) | 4 (2.88) |  |
|  | More frequent | 12 (26.67) | 36 (38.30) | 48 (34.53) |  |
| **Changes in meal times since thyroid removal surgery***^a^* | |  |  |  | .16 |
|  | No change | 41 (91.11) | 77 (81.91) | 118 (84.89) |  |
|  | Staggered schedules | 1 (2.22) | 10 (10.64) | 11 (7.91) |  |
|  | Nocturne | 1 (2.22) | 0 (0) | 1 (0.72) |  |
|  | More frequent | 2 (4.44) | 4 (4.26) | 6 (4.32) |  |
|  | More rarely | 0 (0) | 3 (3.19) | 3 (2.16) |  |
| **Changes in types of food since thyroid removal surgery***^a^* | |  |  |  | **.02** |
|  | No change | 37 (82.22) | 54 (57.45) | 91 (65.47) |  |
|  | Less salty | 4 (8.89) | 23 (24.47) | 27 (19.42) |  |
|  | Less sweet | 1 (2.22) | 6 (6.38) | 7 (5.04) |  |
|  | More acidic | 0 (0) | 1 (1.06) | 1 (0.72) |  |
|  | Saltier | 3 (6.67) | 2 (2.13) | 5 (3.60) |  |
|  | Sweeter | 0 (0) | 8 (8.51) | 8 (5.76) |  |
